# A review of discrete element simulation of ice–structure interaction

**DOI:** 10.1098/rsta.2017.0335

**Published:** 2018-08-20

**Authors:** Jukka Tuhkuri, Arttu Polojärvi

**Affiliations:** Aalto University, Department of Mechanical Engineering, PO Box 14300, Espoo 00076, Finland

**Keywords:** discrete element method, sea ice, marine structures, ships, ice loads, simulation

## Abstract

Sea ice loads on marine structures are caused by the failure process of ice against the structure. The failure process is affected by both the structure and the ice, thus is called ice–structure interaction. Many ice failure processes, including ice failure against inclined or vertical offshore structures, are composed of large numbers of discrete failure events which lead to the formation of piles of ice blocks. Such failure processes have been successfully studied by using the discrete element method (DEM). In addition, ice appears in nature often as discrete floes; either as single floes, ice floe fields or as parts of ridges. DEM has also been successfully applied to study the formation and deformation of these ice features, and the interactions of ships and structures with them. This paper gives a review of the use of DEM in studying ice–structure interaction, with emphasis on the lessons learned about the behaviour of sea ice as a discontinuous medium.

This article is part of the theme issue ‘Modelling of sea-ice phenomena’.

## 1. Introduction

**Figure 1.**
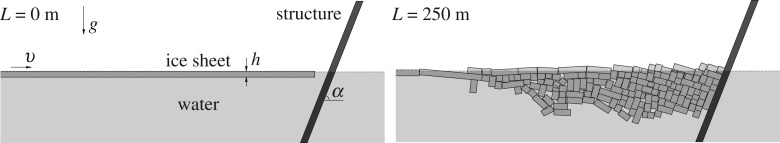
Two snapshots from different stages of a DEM simulation where an intact ice sheet moved from the left against an inclined structure on the right and failed into discrete blocks, which then formed a rubble pile [2]. *L* refers to the amount of ice pushed against the structure.

Sea ice loads on ships and marine structures are caused by the relative movement between the structure and an ice feature, and the sequential failure process of ice. During contact with a structure, sea ice fails into a myriad of small and large pieces that may accumulate into a pile and further affect the ice failure process, and finally, leave the active failure zone. Such an ice failure process has a feedback loop: earlier failure events affect both the initial and the boundary conditions of later ice failure events [1]. As an example, figure 1 illustrates a process where a floating ice sheet is moving against an inclined marine structure and fragmenting from a solid sheet into individual ice blocks forming an ice rubble pile, which will affect the ice failure events later during the process.

Several different ice failure modes have been observed [3–6]. When failing against a marine structure, an ice sheet can fail locally through micro-cracking or flaking and globally through bending or buckling. Important parameters of the structure include inclination, shape, width and stiffness; important parameters of the ice include thickness, salinity, grain size and grain type. The failure process of ice is further affected by the temperature and strain rate; cold ice under fast loading behaves differently than warm ice under slow loading. As the ice failure process is affected by both the ice and the structure, the term *ice–structure interaction* is often used.

Traditionally, the methods used to calculate ice loads have been based on *a priori* assumptions of the ice failure mode. As an example, if we knew that an ice sheet of thickness *h* would fail through bending against a structure with an inclination angle *α* and form a rubble pile with height *H* in front of the structure, and how the rubble would affect the bending failure, we could form equations to estimate the ice load. But we do not know the failure process that well, and maybe we will never know: there is evidence that the failure process is extremely sensitive to initial conditions and we cannot make many *a priori* assumptions of the ice failure mode [7,8].

As the ice load on a marine structure results from a complicated failure process, an effective way to study ice loads is to simulate the ice failure process and to study the properties of the process [1]. Conceptually this approach is not new; process models for ice failure against a vertical pile [9] and against an inclined ship hull [10] have been proposed, but only after the development of modern computers has it been possible to simulate ice failure processes of reasonable length in detail.

Simulations of ice–structure interaction need to consider a complicated process including fragmentation of ice, formation and motion of ice blocks, and interactions between the blocks as well as between the blocks and the structure. One of the numerical methods that can deal with these requirements is the discrete element method (DEM), introduced by Cundall & Strack [11] for modelling the dynamics of systems consisting of individual particles and used in material sciences, geophysics, fracture mechanics and ice mechanics [12–15]. There is also another reason for making DEM well suited for studies on ice–structure interaction: ice appears in nature often as discrete floes; small and large floes floating at the water surface or forming ice ridges.

This paper reviews DEM simulations of the interaction of different ice features with ships and structures. It also reviews DEM modelling of the ice features themselves, as understanding of ice mechanics is needed in understanding the ice loads. The paper starts with an introduction to DEM and closes with a discussion and suggestions for future work.

## 2. Discrete element modelling

**Figure 2.**
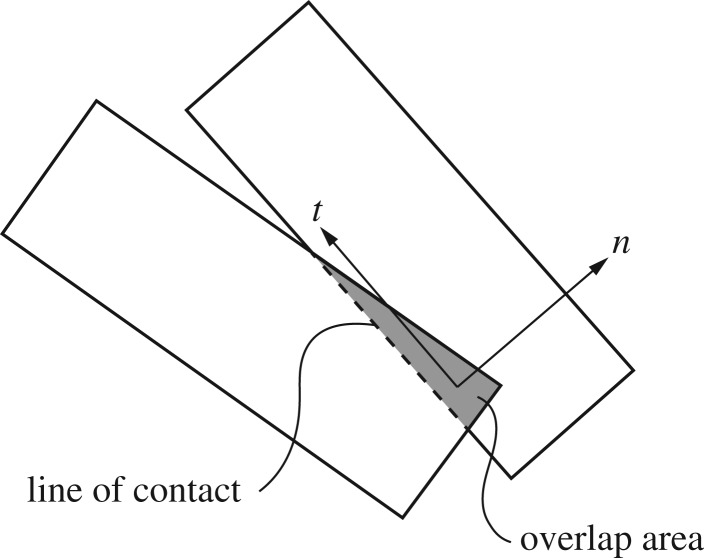
Two polygonal discrete elements in contact. Here, the overlap area (grey) is used to define contact force with normal and tangential components, and the length of the line-of-contact to define a plastic limit for the contact force.

DEM, as formulated by Cundall & Strack [11], has been applied to ice mechanics since the mid-1980s. Typical DEM simulations in ice mechanics consist of a few hundred, up to a few thousand, interacting ice blocks. In the classical DEM formulation, the blocks are rigid, contact forces are solved by using models mimicking the effect of contact deformation, and the simulations are explicit and advance in short time steps. (Recently, implicit, non-smooth and event-driven DEM simulations have also been used [16–21], but these techniques are not described here for brevity.) On a given time step, the contact forces (figure 2) and external forces acting on each block are solved, Newton's second law is used to determine the accelerations, and a numerical integration scheme of choice is used to update the velocities and positions of each block.

Computation of the contact forces is often the bottleneck of DEM simulations. The contact force models allow a very small overlap between blocks in contact and use the overlap geometry, or penetration, to calculate the contact forces (figure 2). Owing to this, the level of detail in describing the ice block shape directly affects the computational cost. The least burdensome shape is a two-dimensional (2D) disc [22–27], but the need for describing the angularity of ice blocks was already recognized in the 1990s [28,29]. Since then, most DEM simulations have used polygons in 2D, and polyhedrons [16,18,30–32] or dilated polyhedrons [33] in 3D to describe ice block shapes. Additionally, ice floes in 3D have been described using discs with finite thickness [34,35].

The contact force has normal and tangential components (figure 2). The normal component is often solved using an elastic–viscous or elastic–viscous–plastic model. In the latter model [12], on each time step the overlap geometry defines an upper limit for the normal force. A viscous load component, solved using the rate of change in the overlap geometry, adds damping into the normal force. Roughly all of the tangential contact force models have their basis on the Coulomb friction model.

The external forces include buoyancy, gravitation and water drag. Implementation of the first two is straightforward, but the models used for drag are crudely simplified [22,36–38]; rigorous modelling of hydrodynamics over even moderate time periods remains unattainable. The most elaborate drag model used is based on potential flow theory [32].

A central part of most ice mechanics simulations is the deformation and failure of ice (figure 1). Deformations have been modelled by using rigid discrete elements joined together with elastic bonds [39] or Timoshenko beams [40], or by using deformable discrete elements [41]. Fractures along element boundaries, and through elements, have been modelled by using Mohr–Coulomb-type criteria in compression [42], maximum tensile strength in bending [43], but also mixed-mode criteria and energy dissipation [40]. For modelling deformation and failure, DEM can be extended into a combined finite element method (FEM)-DEM using finite elements to describe the constitutive behaviour and fracture of ice, and discrete elements to model the contact interactions.

The recent efforts include extension of DEM modelling of ice sheets into 3D by using Timoshenko beams [44] or elastic bonds [18] to join the discrete elements into a sheet. In another approach [31,45], closed-form solutions for the bending failure of a semi-infinite ice sheet on elastic foundation are developed and used to solve a sequence of bending failures in ship–ice interactions. In addition, bonded-particle models, where the ice sheet consists of spheres bonded together by elastic bonds, have been used [46,47].

## 3. Discrete element method simulation of sea ice and ice–structure interaction

### (a) Ice floe fields

Large sea areas are covered with broken ice consisting of individual floes [5]. Ice floe fields are interesting due to the need to understand the dynamics of marginal ice zones, and because shipping favours sea areas with ice floes over packed ice.

**Figure 3.**
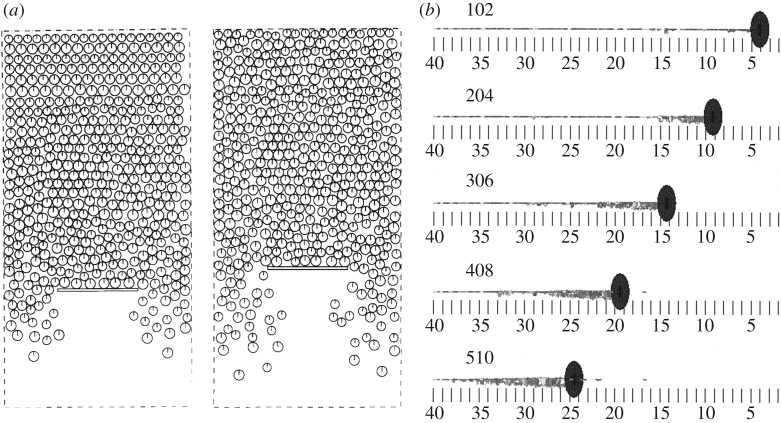
DEM modelling of ice floe fields: (*a*) 2D ice–structure interaction simulations on ice boom loads at two different stages of a simulation [26] and (*b*) vertical snapshots from 3D floe rafting simulations where the pusher moved from the right; note the non-uniform and out-of-plane thickening of the floe field [34].

The early DEM studies on ice floe fields were in 2D and considered systems of circular floes restricted on a plane [22–26,48]. The first study of this kind focused on river ice jams [22] and was followed with studies on floe field dynamics on a larger scale: macroscopic stresses to produce a yield curve and a constitutive model of a floe field [23], clustering of circular floes [27] and dynamics of floes with complex shapes [17]. 2D DEM has also been used to study ice arch formation between river piers [49] and loads on an ice boom dragged through a field of circular floes (figure 3*a*) [25,26]. Experiments with an ice boom showed that even a simple model can yield results that align with experimental results. Simulations of a moored ship in a floe field [50,51] showed only a qualitative agreement with experiments, a result also observed later in similar simulations [52], where the differences were linked with the limitations of 2D modelling and the simplifications with the modelling of hydrodynamics. More recent 2D simulations have studied ships in an ice floe field and suggest that the turning circle is smaller in a floe field than in open water [53,54], that the loads due to ice floe impacts follow a Weibull distribution [55] and the loads on a turret mooring system increase with compression in the ice floe field [56].

However, the horizontal strength of ice jams and ice floe fields also depends on out-of-plane thickening due to floe rafting and floe overturning, which are 3D processes [34,57]. In an early 3D simulation of broken ice, disc-shaped ice floes were confined within a channel and compressed with a plate from one side [34]. It was observed that when the surface concentration of the floes reached 79%, the floe field compression changed from 2D consolidation into 3D rafting and floe overturning. Thus, unless it can be ensured that the floe concentration stays low, the modelling of ice floe fields should be conducted in 3D. These simulations, verified by parallel laboratory tests, also showed the importance of the width of the channel where the floes are: friction at the channel edges affects both the process and the loads; for wide channels, these effects are smaller than for narrow channels. Such 3D DEM simulations have been used to study the interactions of an ice boom and ice floes in a channel [58], the formation of ice jams and forces on structures in rivers [59], and the pancake-ice dynamics in a wave field where it was demonstrated that the thickening due to rafting is a function of wave amplitude, wavelength and floe diameter [60,61]. In all of these studies, the DEM simulations were successfully verified with parallel laboratory experiments and compared with analytical models. 3D DEM has also been used to study ship interaction with ice floes [62–64]. It has been shown how the loads from impacts with ice floes increase with floe concentration and ice thickness [35], how reducing the stiffness of a mooring system decreases ice loads [65], and how the effect of wall constraint on ice resistance is important for ice concentrations over 70% [66]. 3D DEM simulations have further shown that pancake ice loads on a vertical cylinder increase nearly exponentially with wave height, and substantially with ice concentration [67].

### (b) Ice ridges and rubble fields

Ridges are elongated piles of ice blocks and can be tens of metres thick [5]. Rubble field is a term for ridge fields and wide ridges. Ridges form when ice sheets, driven by winds and currents, undergo compression, break into ice blocks and form piles. Understanding of the ridging processes and ridge properties is important: the forces required for ridging limit the global ice loads on marine structures; ridges cause high local ice loads, are major obstacles for shipping, and the energy dissipated during ridging needs to be included in large-scale sea ice models.

In the early 2D DEM simulations, ridges were formed by compressing a layer of floating ice blocks [29] or a layer of ice blocks resting on a frictionless surface [68]. Even the simple simulations of floating ice blocks demonstrated the importance of describing the angularity of ice blocks and that the energy dissipation during ridging is higher than previously assumed. Ridging studies were later extended to model intact ice sheets and their failure into discrete blocks [36,39,43]. In the simulations of ridge formation from thin lead ice compressed between two thick floes, an increase of the friction coefficient of ice decreased the energy dissipation, while the dissipation increased with ice thickness [39]. In further studies, the small-scale results were upscaled to study pack ice dynamics [33,69,70] and used to identify different stages of ridging, and the effects these stages have on ridging loads [71]. However, not all ridges form from lead ice between thick floes, some form from two ice sheets compressed together. 2D DEM simulations of such ridging processes were verified through model-scale experiments [72] and used to study the parameters defining whether ridging or rafting dominates ice sheet deformation [36]. In these parallel experiments and simulations, it was also observed that rafting and ridging are not two different physical processes, but rafting is the precursor to ridging.

Ice rubble, a material comprising discrete ice blocks, has been characterized by properties used in soil mechanics: shear strength, friction angle and cohesion. These properties have been studied experimentally, with varying results [73]. The shear strength of ice rubble has four components [28]: interlocking, frictional contacts, freeze-bonding and failure of the ice blocks. All of these are block-level phenomena and all are affected by the shape of the ice blocks. Simulations of ice rubble deformation with circular and block-shaped particles give different results [29].

**Figure 4.**
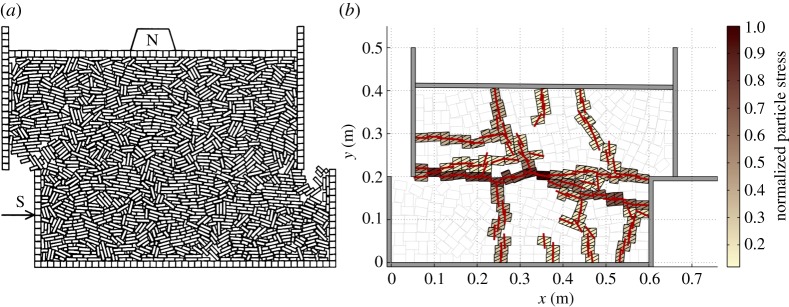
DEM simulations of shear box tests in (*a*) full-scale (box size about 45 m × 30 m) [28] and (*b*) laboratory-scale [74]. The force chains in (*b*) are indicated by colours showing the normalized compressive stress of the blocks.

The early DEM studies on ice rubble modelled shear box experiments (figure 4*a*) [28]. It was observed that the shear strength increased with block-to-block friction, but more interestingly, the shear strength decreased with increasing confining pressure. This decrease was due to interlocking: high confinement hindered the blocks from rearranging, causing them to break instead. It was also suggested that the results of shear box experiments may depend on the experimental set-up. This was confirmed by DEM simulations of laboratory-scale shear box experiments (figure 4*b*): the effect of the experimental apparatus manifested itself through force chains, which ran from wall to wall inside the shear box and defined the peak rubble strength [74].

**Figure 5.**
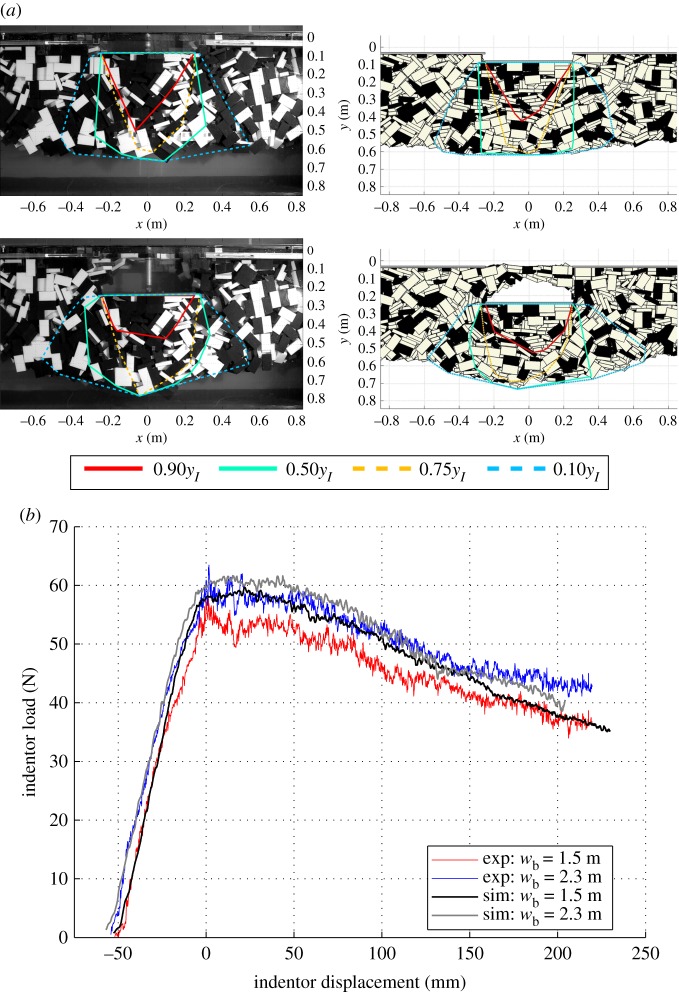
Laboratory-scale punch-through tests and parallel DEM simulations [37]: (*a*) two stages of a flat indentor moving into a floating rubble in experiments (left) and in simulations (right). The coloured lines describe the contours for rubble displacement; *y*_*I*_ is indentor displacement; (*b*) typical indentor load-displacement records from experiments (exp) and simulations (sim) with different basin widths (*w*_b_).

Another type of material test used with ice rubble is the punch-through test [75] where a flat indentor penetrates floating rubble and the indentor load and displacement are measured (figure 5). Punch-through tests have been modelled with circular particles in 2D [76] and 3D [77], and with block-shaped particles in 3D [30]. In experiments, the punch load increases to a peak value at a small displacement, and then slowly decreases. When analysing such tests by using continuum modelling, the peak load is often attributed to cohesion induced by freeze bonds, and the decreasing load, described as material softening, to an advancing failure along a shear plane [78,79]. DEM simulations, however, suggest that there is no unique shear plane within the rubble [30]. Instead, throughout the experiment, the rubble deformation patterns evolve [37]. The evolution of the deformation patterns correlates well with the indentor load and, instead of an advancing shear failure, explains the decrease in the indentor load [80].

The use of DEM to study the interaction between ridges and offshore structures has included modelling ridges as thick areas between thinner level ice [42], load transmission from level ice, through rubble resting on a flat surface, on a structure [81], ridge keel deformation during sea bottom scouring [62], loads on conical structures [82–84] and loads on a vertical cylinder [85]. A 3D study of ridge resistance of a ship [86] explained the relationship between ridge width and resistance.

### (c) Ice sheets

#### (i) Wide inclined structures

Figure 1 illustrates a floating ice sheet failing against an inclined structure. Here, the key engineering questions are the maximum ice load and the maximum height of the rubble pile, both of which can be solved if the ice failure and pile-up processes are correctly modelled. As this process, also called rubbling, includes discrete failure events and a number of interacting ice blocks, it is an archetype of ice engineering problems that can be modelled with DEM, and was one of the first studied [81,87].

The early studies were limited by computer resources and could only analyse the initial part of the process. After the simulations of longer rubbling processes became practical, DEM was used to estimate the ratio of rubbling work to increase in potential energy of the ice blocks, and to discuss the differences between 2D simulations and physical experiments with narrow ice strips [88]. In the simulations, the rubble piles had a lower porosity, and the pile-up process required less work than in the experiments, but in general the forces and failure process were similar. 2D DEM simulations have also been used to study the effects of inclination angle of the structure [89,90] and grounding of the ice [91,92] on the ice failure process.

The failure of an ice sheet against an inclined structure has also been studied with a combined 2D FEM-DEM, where the ice sheet and its fracture are modelled with FEM, while the contact forces between the colliding ice blocks are calculated with DEM [40]. Simulations with this model have provided a number of observations: (i) the failure process includes pile-up, ride-up, shear and pile collapse events [93]; (ii) the most important parameters are the ice thickness and compressive strength, and the inclination angle [8,94,95]; (iii) the importance of parameters change during the failure process [95]; and (iv) the load is transmitted though the rubble by force chains and is limited by buckling of the load chains [2,96]. The model used in these rubbling studies is deterministic, but very sensitive to initial conditions. This allowed the conduction of virtual experiments and creation of data to study the distributions of peak ice loads [8] and the evolution of the ice failure process [97]. The load distributions were right-skewed and thus non-normal. A Gumbel distribution described the data well. Owing to a large scatter, caused by the ice–structure interaction process itself, a large number of observations are needed for studying peak ice load statistics: to observe a 15% difference in peak loads due to a single parameter requires more than 80 peak ice load observations.

#### (ii) Conical offshore structures and ships

The 2D simulations of ice failure against wide inclined structures, discussed above, have provided many interesting results. However, the failure of ice against a conical offshore structure is a 3D process and needs to be simulated as one. The process starts by ice edge crushing and formation of radial cracks until the vertical force is high enough to create circumferential cracks and discrete ice blocks, which pile up against, and clear around, the structure [6]. For ships, the ice failure process is very similar, but ice blocks are also pushed under the bow.

**Figure 6.**
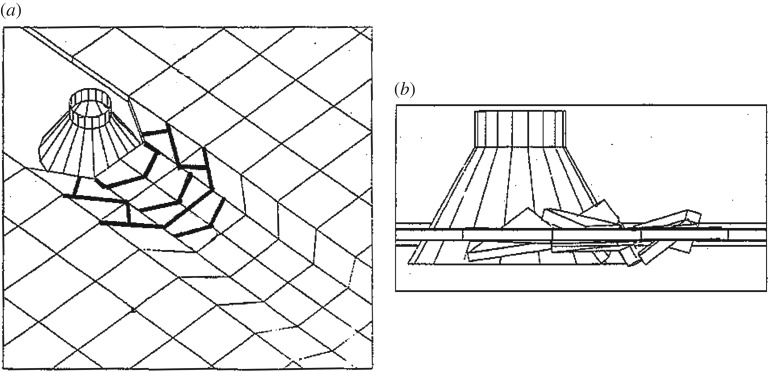
Snapshots from simulations where a floating ice sheet fails against a conical structure: (*a*) the thin lines in the ice sheet show initial element boundaries and the think lines show failure planes; (*b*) side view showing ice pile-up [98].

Both ship–ice and cone–ice (figure 6) interactions have been simulated by using a 3D DEM model of a floating plate that can fail along element boundaries, and along planes through element centroids, to create discrete elements [41]. This model, which uses a Mohr–Coulomb failure criterion, favours fractures through elements over fractures along element boundaries where the mesh defines the fracture directions [41,99]. The model has been used to simulate ice loads on conical structures [98,99], ships turning in ice [100], and the ice resistance of ships [101]. As shown in figure 6, the element size in these simulations has been large and the number of elements small. The elements were not allowed to fracture in all simulations. Clear effects of the domain boundaries were observed at high velocities [101]. However, the simulations with a moored conical structure [99] showed interesting results: (i) depending on the ice velocity, the load increased with increasing stiffness of the mooring system; (ii) the pile size in front of the structure increased with increasing ice thickness; and (iii) the failure process was dominated by bending, with some shear failure events for thick ice with high bending strength, but no tensile failures and, in contrast to field observations, no initial radial cracks were observed, possibly reflecting the way fracturing is simulated in the model.

Bonded-particle models [102], where a solid is represented by spheres bonded together, have been adapted to sea ice sheets. In such models, the stiffness and strength of the spheres and the bonds relate to the stiffness and strength of the ice sheet: the sphere size affects the mechanical behaviour of the modelled ice sheet. Bonded-particle models have been calibrated through simulations of bending and the compressive strength of ice and used to study loads on conical structures [46,47,103,104], ice-induced vibrations of conical structures [105], as well as ice resistance and local loads on ships [106]. While fairly straightforward to implement, the bonded-particle models appear to still be under development: user-specific counter-torque has been added to particles to better model particle rotation, and the sliding of bonded particles against each other is different from the sliding of smooth particles and needs to be tuned [104]. However, it has been shown that bonded-particle models can give similar ice loads to those given by standards [103] and measured in experiments [46], and the models can be used to study shielding effects in multi-leg structures [103].

#### (iii) Vertical structures

Ice failure against a vertical structure, crushing, is a complicated process including contact between ice and the structure, fragmentation of the ice into discrete particles, motion of the particles against and around the structure, and the dynamic response of the structure [1]. The ability to model both the fragmentation of the ice sheet and the motions of ice particles is essential to successful simulation of ice crushing. DEM appears a promising tool to study this process [107,108], but only a few studies have been conducted.

A 2D DEM model, where tensile stresses lead to brittle fragmentation and compressive stresses to viscoplastic flow, has been developed to study the failure of ice against a flexible vertical structure as a plane strain problem [109]. The model includes a property that small ice fragments have a higher strength than large fragments; this restricts the formation of very small particles that would require substantial computational resources, but is also in line with the observed size dependency of ice strength. The results show a fragmentation process of the ice sheet, but do not show the direct line-like contact between the structure and ice, observed in several experiments [1] and in FEM simulations [110]. This may be due to the low ice velocity used. A recent study with a 3D bonded-particle model [111], where the bonds are linearly elastic and failure occurs through tension and shear, has shown formation of high-pressure zones with direct contact with the ice and the structure.

Another class of problems is the impact of an ice floe against a vertical structure. This has been simulated as a plane stress problem, 2D in the horizontal plane. DEM simulations of this case have shown both ice failure in the vicinity of the structure [112] and splitting of the floe [113–115]. It is of practical interest which failure mode occurs and what the roles of different parameters are, including dimensions, velocity and confinement [116].

## 4. Discussion

An ice–structure interaction is a complicated process to model. The ice model must account for large deformations and displacements, fracture and fragmentation, and the motion of the ice fragments formed. For the structure, the model should take into account the static or dynamic response under ice loading, but the structural response may have an effect on how the ice fails; thus this process is called ice–structure interaction. These modelling demands can be dealt with by several different techniques. DEM is one of the suitable methods, especially in applications where the discrete ice blocks have a central role.

The DEM models developed for sea ice applications can be divided into two groups: those where the discrete elements have a physical meaning (an ice floe or an ice block) and those where the discrete elements are used for describing material behaviour and failure in the same way as elements in FEM or lattice models. An example of the former type are models used for ridging, where the formation and interactions of discrete blocks is the key phenomenon, the number of ice blocks is small and the process needs to be modelled as discrete [29]. Examples of the latter type are models for ice crushing and splitting of an ice floe, where the modelling of crack initiation and propagation has a central role. These cases can be modelled with DEM, but also by using other, potentially more effective methods [110].

In other fields such as soil mechanics, continuum models of (macro-scale) material behaviour have been derived based on DEM simulations of particle-level (micro-scale) phenomena. In ice mechanics, this approach has been successfully used to estimate the yield curve of an ice floe field [23]. However, in contrast with soils, where studies concern a large number of particles, many ice mechanics problems involve only a few hundred ice blocks, as illustrated in the figures of this paper, and the applicability of continuum models should be carefully considered. As continuum models are used in ice mechanics, more work for establishing limits for their applicability is needed. Such work can be performed using DEM [117].

Real-time simulation of ship navigation can be useful in crew training and in planning of marine operations. For such simulations, models relying on physics engines and general-purpose computing on graphical processing units have been developed [16,18–21,31,118,119]. Physics engines are software systems dedicated to fast physics-related calculations such as rigid body dynamics, are used in computer games, and may prioritize speed over accuracy. However, it has been shown that a physics engine can succeed in describing the behaviour of a granular material [120]. This encourages the verification and use of physics engines in ice–structure interaction studies. Another computationally effective way to simulate ship–ice interaction is to determine the geometry of ice pieces forming through bending failure numerically, store the results into a database, and then simulate the ship–ice interaction by using the database [121]. Such an approach is limited to the fracture patterns stored in the database.

With increasing computing power and effective numerical techniques, in the future ice failure processes can be modelled in more detail and over longer time periods than today. As ice loads are the result of ice failure processes, simulations of long processes are important. Also, detailed 3D DEM simulations and studies on ice-induced vibrations can be expected to expand in the future. There have been a few DEM simulations on flexible structures and ice-induced vibrations [46,99,105,109], but the challenges in modelling the crushing of ice against structures have kept the number of efforts low. An area requiring more research is the modelling of hydrodynamics in ice–structure interaction. Currently, only simplified models for fluid drag are used, but it is not known how reliably such simple approaches take the hydrodynamics involved into account. For this reason, many simulations are only conducted at low velocities.

DEM has been used for studies on ice–structure interaction since the mid-1980s and major development has taken place since then. However, rather than placing the emphasis on ice mechanics research, many studies have focused on the development of numerical techniques, especially in the case of engineering applications. As the examples in this review demonstrate, careful analysis of DEM-simulated sea ice failure processes has led to several important observations related to the behaviour of sea ice and the physical phenomena behind ice loads. Hopefully, in the future, the use of DEM as an ice mechanics research tool will further expand.

## 5. Conclusion

During the last few decades the DEM has become one of the key numerical methods used to study ice–structure interaction. The review given in this paper has concentrated on DEM as formulated by Cundall & Strack [11]. The emphasis has been on the engineering applications of the method, more than on the development of the method itself. The use of DEM appears most beneficial in cases where modelling of discrete ice blocks is required. It has been observed that:

(i) ice floe fields need to be modelled in 3D, unless it can be ensured that floe concentration stays low everywhere in the simulation domain;
(ii) the possible angularity of ice blocks needs to be modelled. Circular and angular blocks behave in different ways;
(iii) DEM simulations have shown that the energy dissipation during ridging is higher than was previously assumed;
(iv) ice arch formations, or force chains, can be important force-transmitting mechanisms in ice floe fields and in ice rubble, and need to be considered in ice load models;
(v) no unique shear plane can be observed in ice rubble punch-through experiments; and
(vi) DEM simulations can be used to produce ice load data for studying the statistics of ice loading processes.

It can be expected that the use of DEM in research on ice–structure interaction will continue, and important insights will be obtained into the mechanics of local and global ice loads. One active line of research, where computational speed is important, supports the development of ship simulators for ice navigation and the planning of marine operations. The other future challenges include modelling of 3D fragmentation of ice sheets, and the incorporation of hydrodynamics in DEM models.

## Data accessibility

This article has no additional data.

## References

[1] Daley C, Tuhkuri J, Riska K 1998 The role of discrete failures in local ice loads. Cold Reg. Sci. Technol. 27, 197–211. (10.1016/S0165-232X(98)00007-X)

[2] Ranta J, Polojärvi A, Tuhkuri J 2018 Limit mechanisms for ice loads on inclined structures: buckling. Cold Reg. Sci. Technol. 147, 34–44. (10.1016/j.coldregions.2017.12.009)

[3] Sanderson T 1988 Ice mechanics, risks to offshore structures. London, UK/Dordrecht, The Netherlands: Graham & Trotman Inc./Kluwer Academic Publishers Group.

[4] Schulson E, Duval P 2009 Creep and fracture of ice. Cambridge, UK: Cambridge University Press.

[5] Weeks W 2010 On sea ice. Fairbanks, AK: University of Alaska Press.

[6] Palmer A, Croasdale K 2013 Arctic offshore engineering. Singapore: World Scientific Publishing.

[7] Daley C 1992 Ice edge contact and failure. Cold Reg. Sci. Technol. 21, 1–23. (10.1016/0165-232X(92)90002-C)

[8] Ranta J, Polojärvi A, Tuhkuri J 2018 Scatter and error estimates in ice loads—results from virtual experiments. Cold Reg. Sci. Technol. 148, 1–12. (10.1016/j.coldregions.2018.01.002)

[9] Matlock H, Dawkins W, Panak J 1969 A model for the prediction of ice–structure interaction. In *Proc. of the 1st Offshore Technology Conf. 1969, Houston, TX*, vol. 1, pp. 687–694. Houston, TX.

[10] Varsta P, Riska K 1977 Failure process of ice edge caused by impact with a ship's side. In *Ice, Ships and Winter Navigation Symposium*. Oulu University, Oulu, Finland.

[11] Cundall P, Strack O 1979 A discrete numerical model for granular assemblies. Géotechnique 29, 47–65. (10.1680/geot.1979.29.1.47)

[12] Hopkins M 1992 Numerical simulation of systems of multitudinous polygonal blocks. Technical Report 92-22, Cold Regions Research and Engineering Laboratory, CRREL. 69 p.

[13] Munjiza A 2004 The combined finite-discrete element method. Chichester, England: John Wiley & Sons Ltd..

[14] Pöschel T, Schwager T 2005 Computational granular dynamics—models and algorithms. Berlin, Germany: Springer.

[15] O'Sullivan C 2011 Particulate discrete element modelling: a geomechanics perspective. London, UK: Spon Press/Taylor & Francis.

[16] Metrikin I, Løset S 2013 Nonsmooth 3D discrete element simulation of a drillship in discontinuous ice. In *Proc. of the 22nd Int. Conf. on Port and Ocean Engineering under Arctic Conditions, POAC'13*. Espoo, Finland.

[17] Rabatel M, Labbé S, Weiss J 2015 Dynamics of an assembly of rigid ice floes. J. Geophys. Res.: Oceans 120, 5887–5909. (10.1002/2015JC010909)

[18] van den Berg M 2016 A 3-D random lattice model of sea ice. In *Proc. of the Arctic Technology Conf. 2016, St. John's, Canada (ATC)*. Houston, TX.

[19] Daley C, Alawneh S, Peters D, Quinton B, Colbourne B 2012 GPU modeling of ship operations in pack ice. In *Int. Conf. and Exhibition on Performance of Ships and Structures in Ice, ICETECH 2012, Banff, Alberta, Canada*. Alexandria, VA.

[20] Daley C, Alawneh S, Peters D, Colbourne B 2014 GPU-event-mechanics evaluation of ice impact load statistics. In *Arctic Technology Conf.*, OTC 24645. Houston, Texas.

[21] Alawneh S, Dragt R, Peters D, Daley C, Bruneau S 2015 Hyper-real-time ice simulation and modeling using GPGPU. IEEE Trans. Comput. 64, 3475–3487. (10.1109/TC.2015.2409861)

[22] Babic M, Shen H, Bedov G 1990 Discrete element simulations of river ice transport. In *Proc. of the 12th IAHR Int. Symposium on Ice*, vol. 1, pp. 564–574. Espoo, Finland.

[23] Hopkins M, Hibler WD III 1991 Numerical simulations of a compact convergent system of ice floes. Ann. Glaciol. 15, 26–30. (10.1017/S0260305500009502)

[24] Serrer M, Savage S, Sayed M 1993 Visualization of marginal ice zone dynamics. Trans. Inf. Commun. Technol. 5, 363–375.

[25] Løset S 1994 Discrete element modelling of a broken ice field—Part I: model development. Cold Reg. Sci. Technol. 22, 339–347. (10.1016/0165-232X(94)90019-1)

[26] Løset S 1994 Discrete element modelling of a broken ice field—Part II: simulation of ice loads on a boom. Cold Reg. Sci. Technol. 22, 349–360. (10.1016/0165-232X(94)90020-5)

[27] Herman A 2011 Molecular-dynamics simulation of clustering processes in sea-ice floes. Phys. Rev. E - Stat. Nonlinear Soft Matter Phys. 84, 056104 (10.1103/physreve.84.056104) 22181470

[28] Hopkins M, Hibler WD III 1991 On the shear strength of geophysical scale ice rubble. Cold Reg. Sci. Technol. 19, 201–212. (10.1016/0165-232X(91)90009-6)

[29] Hopkins M, Hibler W III, Flato G 1991 On the numerical simulation of the sea ice ridging process. J. Geophys. Res. 96, 4809–4820. (10.1029/90jc02375)

[30] Polojärvi A, Tuhkuri J 2009 3D discrete numerical modelling of ridge keel punch through tests. Cold Reg. Sci. Technol. 56, 18–29. (10.1016/j.coldregions.2008.09.008)

[31] Lubbad R, Løset S 2011 A numerical model for real-time simulation of ship-ice interaction. Cold Reg. Sci. Technol. 65, 111–127. (10.1016/j.coldregions.2010.09.004)

[32] Tsarau A, Lubbad R, Løset S 2014 A numerical model for simulation of the hydrodynamic interactions between a marine floater and fragmented sea ice. Cold Reg. Sci. Technol. 103, 1–14. (10.1016/j.coldregions.2014.03.005)

[33] Hopkins M 2004 Discrete element modeling with dilated particles. Eng. Comput. (Swansea) 21, 422–430. (10.1108/02644400410519866)

[34] Hopkins M, Tuhkuri J 1999 Compression of floating ice fields. J. Geophys. Res. 104, 15 815–15 825. (10.1029/1999jc900127)

[35] Ji S, Li Z, Li C, Shang J 2013 Discrete element modeling of ice loads on ship hulls in broken ice fields. Acta Oceanol. Sin. 32, 50–58. (10.1007/s13131-013-0377-2)

[36] Hopkins M, Tuhkuri J, Lensu M 1999 Rafting and ridging of thin ice sheets. J. Geophys. Res. 104, 13 605–13 613. (10.1029/1999jc900031)

[37] Polojärvi A, Tuhkuri J, Korkalo O 2012 Comparison and analysis of experimental and virtual laboratory scale punch through tests. Cold Reg. Sci. Technol. 81, 11–25. (10.1016/j.coldregions.2012.04.008)

[38] Tsarau A, Lubbad R, Løset S 2016 A numerical model for simulating the effect of propeller flow in ice management. Cold Reg. Sci. Technol. 142, 139–152. (10.1016/j.coldregions.2016.06.002)

[39] Hopkins M 1994 On the ridging of intact lead ice. J. Geophys. Res. 99, 16 351–16 360. (10.1029/94jc00996)

[40] Paavilainen J, Tuhkuri J, Polojärvi A 2009 2D combined finite–discrete element method to model multi-fracture of beam structures. Eng. Comput. (Swansea) 26, 578–598. (10.1108/02644400910975397)

[41] Hocking G 1992 The discrete element method for analysis of fragmentation of discontinua. Eng. Comput. (Swansea) 9, 145–155. (10.1108/eb023854)

[42] Hocking G, Mustoe G, Williams J 1985 Validation of the CICE code for ice ride-up and ice ridge cone interaction. In *Proc. of the Conf. Arctic'85 Civil Engineering in the Arctic Offshore, San Francisco, CA*, pp. 962–970. New York, NY: ASME.

[43] Hopkins M, Hibler W III 1991 On the ridging of a thin sheet of lead ice. Ann. Glaciol. 15, 81–86. (10.1017/S0260305500009575)

[44] Paavilainen J, Polojärvi A, Tuhkuri J 2010 Jatkuvan murtumisprosessin mallinnus jää-rakenne vuorovaikutuksessa. Technical report, Technical Research Centre of Finland.

[45] Su B, Riska K, Moan T 2010 A numerical method for the prediction of ship performance in level ice. Cold Reg. Sci. Technol. 60, 177–188. (10.1016/j.coldregions.2009.11.006)

[46] Ji S, Di S, Liu S 2015 Analysis of ice load on conical structure with discrete element method. Eng. Comput. (Swansea) 32, 1121–1134. (10.1108/ec-04-2014-0090)

[47] Morgan D, Sarracino R, McKenna R, Thissen J 2015 Simulations of ice rubbling against conical structures using 3D DEM. In *Proc. of the 23rd Int. Conf. on Port and Ocean Engineering under Arctic Conditions, POAC'15*. Trondheim, Norway.

[48] Gutfraind R, Savage S 1997 Marginal ice zone rheology: comparison of results from continuum-plastic models and discrete-particle simulations. J. Geophys. Res. 102, 12 647–12 661. (10.1029/97jc00124)

[49] Schacter M, Spencer D 1994 Parameters influencing ice arch formation. In *The Proc. of 12th IAHR Int. Symposium on Ice*. Trondheim, Norway.

[50] Hansen E, Løset S 1999 Modelling floating offshore units moored in broken ice: model description. Cold Reg. Sci. Technol. 29, 97–106. (10.1016/S0165-232X(99)00023-3)

[51] Hansen E, Løset S 1999 Modelling floating offshore units moored in broken ice: comparing simulations with ice tank tests. Cold Reg. Sci. Technol. 29, 107–119. (10.1016/S0165-232X(99)00017-8)

[52] Karulin E, Karulina M 2011 Numerical and physical simulations of moored tanker behaviour. Ships Offshore Struct. 6, 179–184. (10.1080/17445302.2010.544087)

[53] Zhan D, Agar D, He M, Spenced D, Molyneux D 2010 Numerical simulation of ship maneuvering in pack ice. In *Proc. of the Int. Conf. on Ocean, Offshore and Arctic Engineering, OMAE 2010, Shanghai, China,* vol. 4, pp. 855–862. New York, NY.

[54] Kim H, Sawamura J 2016 A simulation study on the turning ability of ice-going ship navigating in pack ice. In *Proc. of 23th IAHR Int. Symposium on Ice*. Ann Arbor, Michigan, USA.

[55] Han Y, Sawamura J 2017 Fatigue damage calculation for ship hulls operating in pack ice. In *Proceedings of the 24th International Conference on Port and Ocean Engineering under Arctic Conditions, POAC'17*. Busan, Korea.

[56] Karulin E, Karulina M 2017 Environmental effects on dynamic behavior of moored turret ship based on numerical simulations. In *Proc. of the 24th Int. Conf. on Port and Ocean Engineering under Arctic Conditions, POAC'17*. Busan, Korea.

[57] McKenna R, Spencer D, Lau M, Walker D, Crocker G 1997 Modelling the forces exerted by pack ice consisting of small floes. In *Proc. of the 16th Int. Conf. on Offshore Mechanics and Arctic Engineering, OMAE 1997, Yokohama, Japan,* volume IV, pp. 329–338. New York, NY.

[58] Hopkins M, Tuthill A 2002 Ice boom simulations and experiments. J. Cold Regions Eng. 16, 138–155. (10.1061/(ASCE)0887-381X(2002)16:3(138)

[59] Hopkins M, Daly S 2003 Recent advances in discrete element modeling of river ice. In *12th workshop on the hydraulics of ice covered rivers*. Edmonton, Canada.

[60] Hopkins M, Shen H 2001 Simulation of pancake-ice dynamics in a wave field. Ann. Glaciol. 33, 355–360. (10.3189/172756401781818527)

[61] Dai M, Shen H, Hopkins M, Ackley S 2004 Wave rafting and the equilibrium pancake ice cover thickness. J. Geophys. Res. 109 (10.1029/2003jc002192)

[62] Lau M, Lawrence K, Rothenburg L 2011 Discrete element analysis of ice loads on ships and structures. Ships Offshore Struct. 6, 211–221. (10.1080/17445302.2010.544086)

[63] Vroegrijk E 2012 Application of the discrete element method (DEM) on ship-ice interaction. In *Int. Conf. and Exhibition on Performance of Ships and Structures in Ice, ICETECH 2012, Banff, Alberta, Canada.* Alexandria, VA.

[64] Zhan D, Molyneux D 2012 3-dimensional numerical simulation of ship motion in pack ice. In *Proc. of the Int. Conf. on Ocean, Offshore and Arctic Engineering, OMAE 2012, Rio de Janeiro, Brazil,* vol. 6, pp. 407–414. New York, NY.

[65] Molyneux D, Liu L, Zhan D, Reid G 2012 Ice loads on a moored floating production unit. In *Int. Conf. and Exhibition on Performance of Ships and Structures in Ice, ICETECH 2012, Banff, Canada,* pp. 298–304. Alexandria, VA.

[66] Lau M, Sim oes Ré A 2006 Performance of survival craft in ice environments. In *7th Int. Conf. and Exhibition on Performance of Ships and Structures in Ice, ICETECH 2006, Banff, Canada,* pp. 51–58. Alexandria, VA.

[67] Sun S, Shen H 2012 Simulation of pancake ice load on a circular cylinder in a wave and current field. Cold Reg. Sci. Technol. 78, 31–39. (10.1016/j.coldregions.2012.02.003)

[68] Evgin E, Frederking R, Zhan C 1992 Distinct element modeling of ice ridge formation. In *The Proc. of the 11th Int. Conf. on Offshore Mechanics and Arctic Engineering, OMAE 1992, Calcagy, Canada,* vol. IV, pp. 255–260. New York, NY.

[69] Hopkins M 1996 On the mesoscale interaction of lead ice and floes. J. Geophys. Res.: Oceans 101, 18 315–18 326. (10.1029/96JC01689)

[70] Hopkins M, Thorndike A 2006 Floe formation in arctic sea ice. J. Geophys. Res.: Oceans 111 (10.1029/2005jc003352)

[71] Hopkins M 1998 Four stages of pressure ridging. J. Geophys. Res. 103, 21 883–21 891. (10.1029/98jc01257)

[72] Tuhkuri J, Lensu M 2002 Laboratory tests on ridging and rafting of ice sheets. J. Geophys. Res. 107, 13 605–13 613. (10.1029/2001jc000848)

[73] Ettema R, Urroz G 1989 On internal friction and cohesion in unconsolidated ice rubble. Cold Reg. Sci. Technol. 16, 237–247. (10.1016/0165-232X(89)90025-6)

[74] Polojärvi A, Pustogvar A, Tuhkuri J 2015 DEM simulations of direct shear box experiments of ice rubble: force chains and peak loads. Cold Reg. Sci. Technol. 116, 12–23. (10.1016/j.coldregions.2015.03.011)

[75] Leppäranta M, Hakala R 1992 The structure and strength of first-year ice ridges in the Baltic sea. Cold Reg. Sci. Technol. 20, 295–311. (10.1016/0165-232X(92)90036-T)

[76] Karulin E, Karulina M 2002 Simulation of ridge keel behaviour in direct shear and punch tests by discrete element method. In *Proc. of the 16th IAHR Int. Symposium on Ice*, vol. 3, pp. 143–151. Dunedin, New Zealand.

[77] Sorsimo A, Heinonen J 2014 Modelling ice ridge punch tests with cohesive 3D discrete element method. In *Proc. of the 22nd IAHR Int. Symposium on Ice*. Singapore.

[78] Heinonen J 2004 Constitutive modeling of ice rubble in first-year ridge keel. Doctoral Thesis, TKK. VTT Publications 536. Espoo, Finland.

[79] Serré N 2011 Mechanical properties of model ice ridge keels. Cold Reg. Sci. Technol. 67, 89–106. (10.1016/j.coldregions.2011.02.007)

[80] Polojärvi A, Tuhkuri J 2013 On modeling cohesive ridge keel punch through tests with a combined finite-discrete element method. Cold Reg. Sci. Technol. 85, 191–205. (10.1016/j.coldregions.2012.09.013)

[81] Evgin E, Zhan C, Timco G 1992 Distinct element modeling of load transmission through grouded ice rubble. In *The Proc. of the 11th Int. Conf. on Offshore Mechanics and Arctic Engineering, OMAE 1992, Calcagy, Canada,* vol. IV, pp. 273–279. New York, NY.

[82] Haase A, Polojärvi A, Tuhkuri J 2010 3D discrete numerical modelling of conical structure-ice rubble interaction. In *The Proc. of 20th IAHR Int. Symposium on Ice, 2010, Lahti, Finland*. Helsinki, Finland.

[83] Molyneux D, Liu L, Cholley JE 2012 Numerical prediction of first year ice ridge loads on floating offshore structures. In *Arctic Technology Conf.*, OTC 23758. Houston, Texas.

[84] Yulmetov R, Bailey E, Ralph F 2017 A discrete element model of ice ridge interaction with a conical structure. In *Proc. of the 24th Int. Conf. on Port and Ocean Engineering under Arctic Conditions, POAC'17*. Busan, Korea.

[85] Molyneux D, Spencer D, Liu L 2013 Loads due to first year ice ridges on a vertical cylinder. In *Proc. of the 32nd Int. Conf. on Ocean, Offshore and Arctic Engineering, OMAE 2013, Nantes, France*. New York, NY.

[86] Gong H, Polojärvi A, Tuhkuri J 2017 Preliminary 3D DEM simulations on ridge keel resistance on ships. In *Proc. of the 24th Int. Conf. on Port and Ocean Engineering under Arctic Conditions, POAC'17*. Busan, Korea.

[87] Hocking G, Mustoe G, Williams J 1985 Influence of artifical island side-slopes on ice ride-up and pile-up. In *Proc. of the Conf. Arctic '85 Civil Engineering in the Arctic Offshore, San Francisco, CA,* pp. 185–192. New York, NY: ASME.

[88] Hopkins M 1997 Onshore ice pile-up: a comparison between experiments and simulations. Cold Reg. Sci. Technol. 26, 205–214. (10.1016/S0165-232X(97)00015-3)

[89] Li C, Wang Y, Sun H, Li Z 2004 The simulation sea ice pile-up on inclined structure. In *Proc. of the 17th IAHR Int. Symposium on Ice*, vol. 1, pp. 23–30. Saint Petersburg, Russia.

[90] Li C, Wang Y, Li Z, Sun H, Li H 2007 The simulation sea ice pile-up on a semicircle structure. In *POAC'07*, vol. 1, pp. 315–327. Dalian, China.

[91] Goldstein R, Onishchenko D, Osipenko N, Shushpannikov P, Naumov M 2013 Grounded ice pile-up. 2D DEM simulation. In *The Proc. of the 22nd Int. Conf. on Port and Ocean Engineering under Arctic Conditions, POAC'13*. Espoo, Finland.

[92] Polojärvi A, Tuhkuri J, Schneider S, Häsä R 2016 2D FEM-DEM study on ice loads on shallow water structure. In *Proceedings of the 23th IAHR International Symposium on Ice*. Ann Arbor, Michigan, USA.

[93] Paavilainen J, Tuhkuri J, Polojärvi A 2011 2D numerical simulations of ice rubble formation process against an inclined structure. Cold Reg. Sci. Technol. 68, 20–34. (10.1016/j.coldregions.2011.05.003)

[94] Paavilainen J, Tuhkuri J 2012 Parameter effects on simulated ice rubbling forces on a wide sloping structure. Cold Reg. Sci. Technol. 81, 1–10. (10.1016/j.coldregions.2012.04.005)

[95] Ranta J, Polojärvi A, Tuhkuri J 2016 The statistical analysis of peak ice loads in a simulated ice-structure interaction process. Cold Reg. Sci. Technol. 133, 46–55. (10.1016/j.coldregions.2016.10.002)

[96] Paavilainen J, Tuhkuri J 2013 Pressure distributions and force chains during simulated ice rubbling against sloped structures. Cold Reg. Sci. Technol. 85, 157–174. (10.1016/j.coldregions.2012.09.005)

[97] Ranta J, Polojärvi A, Tuhkuri J 2018 Ice loads on inclined marine structures—virtual experiments on ice failure process evolution. Mar. Struct. 57, 72–86. (10.1016/j.marstruc.2017.09.004)

[98] Lau M 2001 A three dimensional discrete element simulation of ice sheet impacting a 60° conical structure. In *Proc. of the 16th Int. Conf. on Port and Ocean Engineering under Arctic Conditions, POAC'01*, vol. 1, pp. 431–440. Ottawa, Canada.

[99] Lawrence K 2009 Load prediction for a moored conical drillship in level unbroken ice: a discrete element and experimental investigation. PhD thesis, University of Waterloo.

[100] Lau M 2006 Discrete element modeling of ship manoeuvring in ice. In *Proc. of the 18th IAHR Int. Symposium on Ice*, vol. 2, pp. 25–32. Sapporo, Japan.

[101] Molyneux W, Spencer D, Liu L, Zhan D 2012 Simulation of ship performance in ice using a discrete element method. In *The ice class ships*. London, UK: The Royal Institution of Naval Architects.

[102] Potyondy D, Cundall P 2004 A bonded-particle model for rock. Int. J. Rock Mech. Mining Sci. 41, 1329–1364. (10.1016/j.ijrmms.2004.09.011)

[103] Di S, Xue Y, Wang Q, Bai X 2017 Discrete element simulation of ice loads on narrow conical structures. Ocean Eng. 146, 282–297. (10.1016/j.oceaneng.2017.09.033)

[104] Morgan D 2016 An improved three-dimensional discrete element model for ice-structure interaction. In *Proc. of the 23rd IAHR Int. Symposium on Ice*. Ann Arbor, Michigan, USA.

[105] Wang S, Ji S 2016 Analysis of ice-induced vibration of conical jacket platform with a coupled discrete-finite element method. In *Proc. of the 23rd IAHR Int. Symposium on Ice*. Ann Arbor, Michigan, USA.

[106] Chen X, Ji S 2014 Discrete element modelling of interaction between level ice and ship hull. In *Proc. of 22nd IAHR Int. Symposium on Ice*. Singapore.

[107] Hocking G, Williams J, Mustoe G 1985 Dynamic global forces on an offshore structure from multi-year ice floe impacts. In *Proc. of the Conf. Arctic'85 Civil Engineering in the Arctic Offshore, San Francisco, CA,* pp. 202–210. New York, NY: ASME.

[108] Sepehr K, Selvadurai A, Comfort G 1997 Discrete element modelling of the local interaction between a stationary structure and a moving ice pack. In *Proc. of the Seventh Int. Offshore and Polar Engineering Conference, 1997, Honolulu, USA,* vol. II, pp. 480–486. Cupertino, CA.

[109] Selvadurai A, Sepehr K 1999 Two-dimensional discrete element simulations of ice-structure interaction. Int. J. Solids Struct. 3, 4919–4940. (10.1016/s0020-7683(98)00272-8)

[110] Kuutti J, Kolari K, Marjavaara P 2013 Simulation of ice crushing experiments with cohesive surface methodology. Cold Reg. Sci. Technol. 92, 17–28. (10.1016/j.coldregions.2013.03.008)

[111] Long X, Ji S 2017 The attributes of local ice pressure analyzed by discrete element method. In *Proc. of the 24th Int. Conf. on Port and Ocean Engineering under Arctic Conditions, POAC'17*. Busan, Korea.

[112] Selvadurai A 2009 Fragmentation of ice sheets during impact. Comput. Model. Eng. Sci. 52, 259–277.

[113] Jirásek M, Bažant Z 1995 Particle model for quasibrittle fracture and application to sea ice. J. Eng. Mech. 121, 1016–1025. (10.1061/(ASCE)0733-9399(1995)121:9(1016))

[114] Kioka S, Yamamoto Y, Mori M, Takeuchi T 2009 Medium-scale test and numerical simulation using dem for the impact load by a high speed ice floe against a structure. In *Proc. of the 20th Int. Conf. on Port and Ocean Engineering under Arctic Conditions, POAC'09*. Luleå, Sweden.

[115] Kioka S, Yamamoto Y, Sugawara K, Endo T, Takeuchi T 2010 Medium-scale experiment and numerical simulation using 3-d dem for the impact load by an ice floe against a pile structure. In *Proc. of the 20th IAHR Int. Symposium on Ice, 2010, Lahti, Finland*. Helsinki, Finland.

[116] Lu W, Lubbad R, Løset S 2015 In-plane fracture of an ice floe: a theoretical study on the splitting failure mode. Cold Reg. Sci. Technol. 110, 77–101. (10.1016/j.coldregions.2014.11.007)

[117] Kulyakhtin S, Polojärvi A 2017 Variation of stress in virtual biaxial compression test of ice rubble. In *Proc. of the 24th Int. Conf. on Port and Ocean Engineering under Arctic Conditions, POAC'17*. Busan, Korea.

[118] Konno A 2009 Resistance evaluation of ship navigation in brash ice channels with physically based modeling. In *Proc. of the 20th Int. Conf. on Port and Ocean Engineering under Arctic Conditions, POAC'09*. Luleå, Sweden.

[119] Metrikin I, Gürtner A, Bonnemaire B, Tan X, Fredriksen A, Sapelnikov D 2015 SIBIS: a numerical environment for simulating offshore operations in discontinuous ice. In *Proc. of the 23rd Int. Conf. on Port and Ocean Engineering under Arctic Conditions, POAC'15*. Trondheim, Norway.

[120] Pytlos M, Gilbert M, Smith CC 2015 Modelling granular soil behaviour using a physics engine. Géotech. Lett. 5, 243–249. (10.1680/jgele.15.00067)

[121] Sawamura J 2012 Numerical investigation of ice bending failure and ice submerging force for ship maneuvering in level ice. In *Proc. of the 20th IAHR Int. Symposium on Ice*, pp. 1116–1128. Dalian, China. 10.1155/2012/301894 PMC3235502 22191063

